# Phylogenetic Gaussian Process Model for the Inference of Functionally Important Regions in Protein Tertiary Structures

**DOI:** 10.1371/journal.pcbi.1003429

**Published:** 2014-01-16

**Authors:** Yi-Fei Huang, G. Brian Golding

**Affiliations:** Department of Biology, McMaster University, Hamilton, Ontario, Canada; University of California San Diego, United States of America

## Abstract

A critical question in biology is the identification of functionally important amino acid sites in proteins. Because functionally important sites are under stronger purifying selection, site-specific substitution rates tend to be lower than usual at these sites. A large number of phylogenetic models have been developed to estimate site-specific substitution rates in proteins and the extraordinarily low substitution rates have been used as evidence of function. Most of the existing tools, e.g. Rate4Site, assume that site-specific substitution rates are independent across sites. However, site-specific substitution rates may be strongly correlated in the protein tertiary structure, since functionally important sites tend to be clustered together to form functional patches. We have developed a new model, GP4Rate, which incorporates the Gaussian process model with the standard phylogenetic model to identify slowly evolved regions in protein tertiary structures. GP4Rate uses the Gaussian process to define a nonparametric prior distribution of site-specific substitution rates, which naturally captures the spatial correlation of substitution rates. Simulations suggest that GP4Rate can potentially estimate site-specific substitution rates with a much higher accuracy than Rate4Site and tends to report slowly evolved regions rather than individual sites. In addition, GP4Rate can estimate the strength of the spatial correlation of substitution rates from the data. By applying GP4Rate to a set of mammalian B7-1 genes, we found a highly conserved region which coincides with experimental evidence. GP4Rate may be a useful tool for the *in silico* prediction of functionally important regions in the proteins with known structures.

## Author Summary

To understand how a protein functions, a critical step is to know which regions in its protein tertiary structure may be functionally important. Functionally important protein regions are typically more conserved than other regions because mutations in these regions are more likely to be deleterious. A number of phylogenetic models have been developed to identify conserved sites or regions in proteins by comparing protein sequences from multiple species. However, most of these methods treat amino acid sites independently and do not consider the spatial clustering of conserved sites in the protein tertiary structure. Therefore, their power of identifying functional protein regions is limited. We develop a new statistical model, GP4Rate, which combines the information from the protein sequences and the protein tertiary structure to infer conserved regions. We demonstrate that GP4Rate outperforms Rate4Site, the most widely used phylogenetic software for inferring functional amino acid sites, via simulations with a case study of B7-1 genes. GP4Rate is a potentially useful tool for guiding mutagenesis experiments or providing insights on the relationship between protein structures and functions.

## Introduction

An important question in biology is the identification of functional residues in proteins. This information can help us understand the relationship between protein structures and functions as well as guide us to design new proteins by genetic engineering. However, experimental techniques for identifying functional sites, e.g. mutagenesis, are time consuming and expensive, which prohibits the brute force scanning of functional sites by experiments. Therefore, bioinformatics tools are useful, because they can narrow down the candidate sites for experimental investigation. Evolution operates similar to a high-throughput mutagenesis experiment: spontaneous mutations introduce protein variants in each generation and then the functional effects of the spontaneous mutations are “measured” by natural selection [1]. Therefore, protein sequences contain signatures of natural selection which reflect the functions of amino acid residues. For example, mutations at the functionally important sites tend to disrupt the proteins' normal functions, so these sites usually are more conserved than unimportant ones. If the sequences of a family of homologous proteins can be collected from multiple species, we may compare these sequences to infer which sites are more important than others.

A number of bioinformatics tools based on phylogenetics have been developed to infer functional sites by the simple idea that functionally important amino acid sites tend to be more conserved than unimportant ones [2]–[11]. Given the multiple sequence alignment and the phylogenetic tree of a protein family, these phylogenetic methods can infer the amino acid substitution rate at each site in the alignment and an unusually low substitution rate implies that the site is functionally important. It has been shown that the predicted conserved sites coincide with experimental evidence, which confirms that these bioinformatics tools are useful.

However, these existing methods are far from flawless. Most of the popular methods, e.g. Rate4Site [7] used in the ConSurf web server [11], assume that the substitution rates are independent across sites. In statistical terms, this means that the sites in the alignment are independent and identically distributed (i.i.d.). The i.i.d. assumption simplifies the statistical modeling, but it is unrealistic from the viewpoint of biology. The i.i.d. assumption implies that the slowly evolved functional sites are randomly distributed in the protein tertiary structure. In contrast, it is well known that functionally important sites tend to be close to each other in the protein tertiary structure and form functional regions, e.g. ligand binding sites or catalytic active sites. Clearly the i.i.d. assumption is inappropriate if a functional region consists of a number of sites.

Several methods have been developed to incorporate the spatial correlation of evolutionary patterns, e.g. substitution rates at the protein level or *dN/dS* ratios at the codon level, to overcome the drawbacks of the i.i.d. assumption [3], [5], [8], [12]–[16]. Most of these methods use a sliding window framework, in which the amino acid substitution rate or the *dN/dS* ratio at a focal site is approximated by the average substitution rate in a set of neighbor sites in the protein tertiary structure [3], [12], [13]. A site is considered to be a neighbor of the focal site if the Euclidean distance between the two sites is smaller than a predefined window size. Unfortunately, these sliding window methods also have intrinsic drawbacks. Firstly, in most, if not all, of sliding window methods the neighbor sites, including the focal site itself, are weighted equally in the inference of the substitution rate. However, clearly the focal site itself contains more information on its substitution rate than the sites near the boundary of the sliding window. Secondly, it is unclear how to determine the optimal window size [17], [18]. If the window size is too large, there will be too many distant sites in the window, which could bias the estimation at the focal site. In contrast, if the window size is too small, the sliding window methods will not be able to capture the spatial correlation of substitution rates and may lead to overfitting. Furthermore, there is evidence that the optimal window sizes may vary among different protein families [12].

Very recently, a Bayesian model which combines the Potts model in statistical physics and the phylogenetic model has been proposed by Watabe and Kishino to infer protein patches under positive selection in protein tertiary structures [16]. In Watabe and Kishino's model, the Potts model is used to define a prior distribution of *dN/dS* ratios over a protein tertiary structure. This model solved many problems of the sliding window framework. However, the prior distribution in Watabe and Kishino's model is unnormalized [16], which makes it difficult to design efficient algorithms to estimate hyperparameters. An advanced algorithm, thermodynamic integration [19], was used in Watabe and Kishino's model to infer hyperparameters. However, the algorithm may be very inefficient, especially if there are many hyperparameters in the Potts model.

Here we propose to incorporate a Gaussian process with the phylogenetic model to overcome the drawbacks of the existing methods. The Gaussian process has been widely applied in geostatistics and machine learning to capture the spatial correlation of interesting features [20], [21]. Here we will briefly introduce the basic idea of the Gaussian process. More details of the Gaussian process and its applications can be found in the geostatistics and machine learning literature, e.g. [20]. A Gaussian process defines a probability distribution over functions, namely that a single sample point of the Gaussian process is a function over a space, e.g. a 3D space. Because the sample points of the Gaussian process are “smooth” functions, the Gaussian process encodes an intrinsic spatial correlation. Thus physically closely located points in the space are more likely to have similar function values. Therefore, the Gaussian process is very useful for defining prior distributions over spatially correlated patterns. For example, in this paper we are interested in modeling the spatial correlation of site-specific substitution rates in protein tertiary structures. If we image each residue in a protein tertiary structure as a single point in the 3D space, the Gaussian process can be used to define a prior distribution of site-specific log substitution rates over these points (residues). The “smoothness” property of Gaussian process prior suggests that two physically closely located sites are more likely to have similar site-specific log substitution rates than two distantly located sites. Then, the Gaussian process prior can be combined with standard phylogenetic likelihood functions [22] to infer site-specific substitution rates from real data.

We name this kind of hybrid model of Gaussian processes and phylogenetics as a phylogenetic Gaussian process model (Phylo-GPM). In the Phylo-GPM framework, the spatial correlation of substitution rates can be naturally described and the strength of spatial correlation can be learned from the data. Therefore, it overcomes the common drawback of the sliding window methods that the window size must be manually specified. Unlike Watabe and Kishino's model [16], the phylogenetic Gaussian process model uses a normalized prior, so simple algorithms, i.e. the widely used Metropolis algorithm [23], [24], can be used to efficiently infer hyperparameters. We have developed software, GP4Rate, based on the Phylo-GPM framework. In both simulated and real datasets, GP4Rate outperforms Rate4Site, a widely used tool based on the i.i.d. assumption. Therefore, GP4Rate may be a useful tool for the identification of functionally important sites.

## Results

### 2D toy protein simulations

Simulations were implemented to evaluate the performance of GP4Rate and to compare it with the widely used software, Rate4Site [7]. In the comparisons, Rate4Site is used as a representative of the classic phylogenetic models which use the discrete Gamma distribution to describe the variation of substitution rates across sites [25] but do not consider the spatial correlation of site-specific substitution rates in the protein tertiary structure. Because the true site-specific substitution rates are known in the simulated alignments, the estimated site-specific substitution rates can be compared with the true rates to evaluate the performance of the two methods. We generated two sets of simulated alignments based on different assumptions. In this and the next section, we will describe the first set of simulations which were based on a 2D toy protein structure. Thereafter we will describe the second set of simulations which were based on more realistic assumptions.

**Figure 1:**
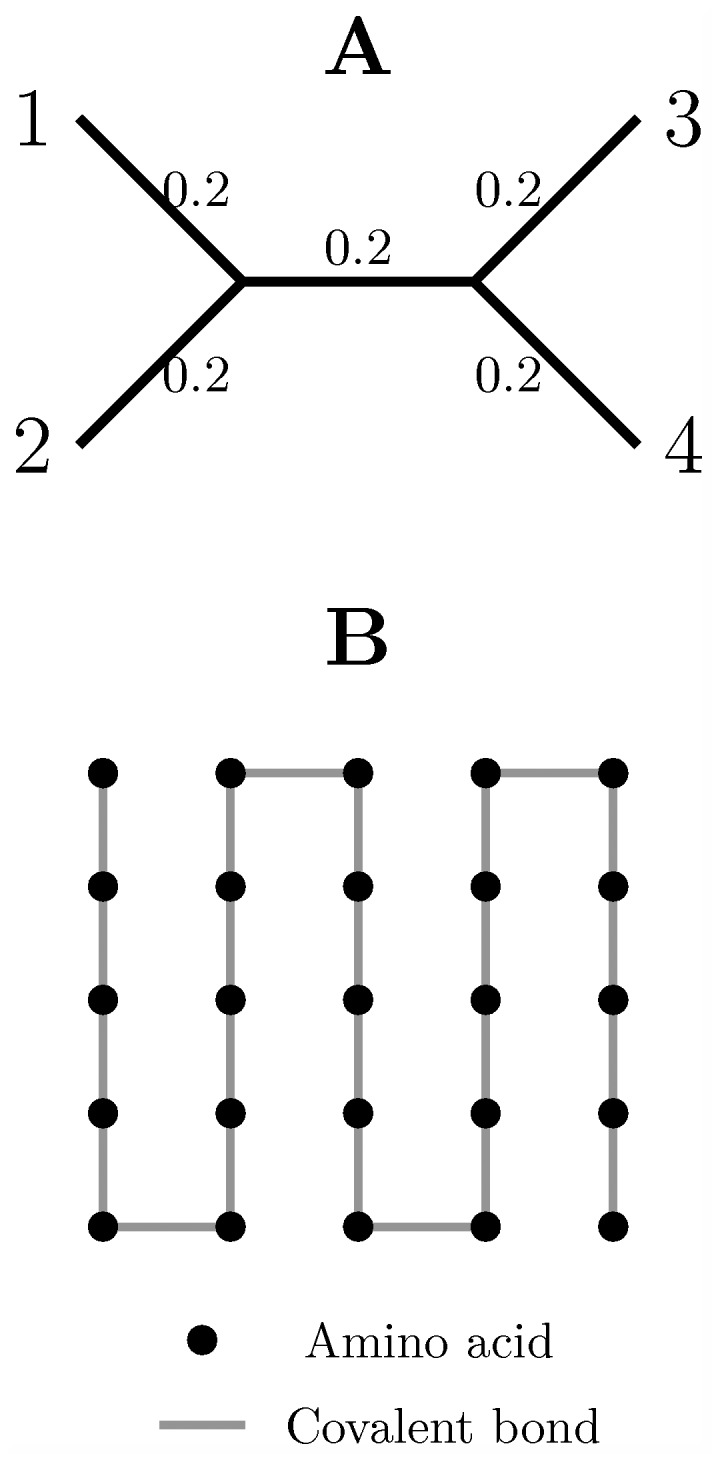
The phylogenetic tree used in all simulations and an example of 2D toy protein structure. (A) the phylogenetic tree used in all simulations; (B) a 5 by 5 2D toy protein. In the phylogenetic tree, there are 4 species and all branch lengths are equal to 0.2 substitutions per site. In the example of 2D toy protein, there are 25 amino acids which are dots in a 5 by 5 2D grid. Lines between dots correspond to the “covalent bonds” between amino acid residues. A larger 20 by 20 2D toy protein with 400 residues is used in the 2D toy protein simulations.

To generate simulated alignments, we need a phylogenetic tree to describe the evolutionary relationship between simulated sequences, a protein structure to calculate the pairwise Euclidean distances between sites, a substitution model, and a vector of substitution rates. Note that the following discussions will be mainly based on the substitution rates rather than their log values. A simple phylogenetic tree was used in all simulations (Figure 1A). The tree consisted of four sequences and all the branch lengths were equal to 0.2 substitution per site. Because the total branch length was equal to 1 substitution per site, on average an amino acid site only contained a single substitution. Therefore, the accurate estimation of substitution rate at a single site is challenging. The JTT substitution model [26], [27] was used in all simulations. Note that the protein tertiary structure and the vectors of substitution rates used in the two sets of simulated alignments were different and will be described in detail below.

**Figure 2:**
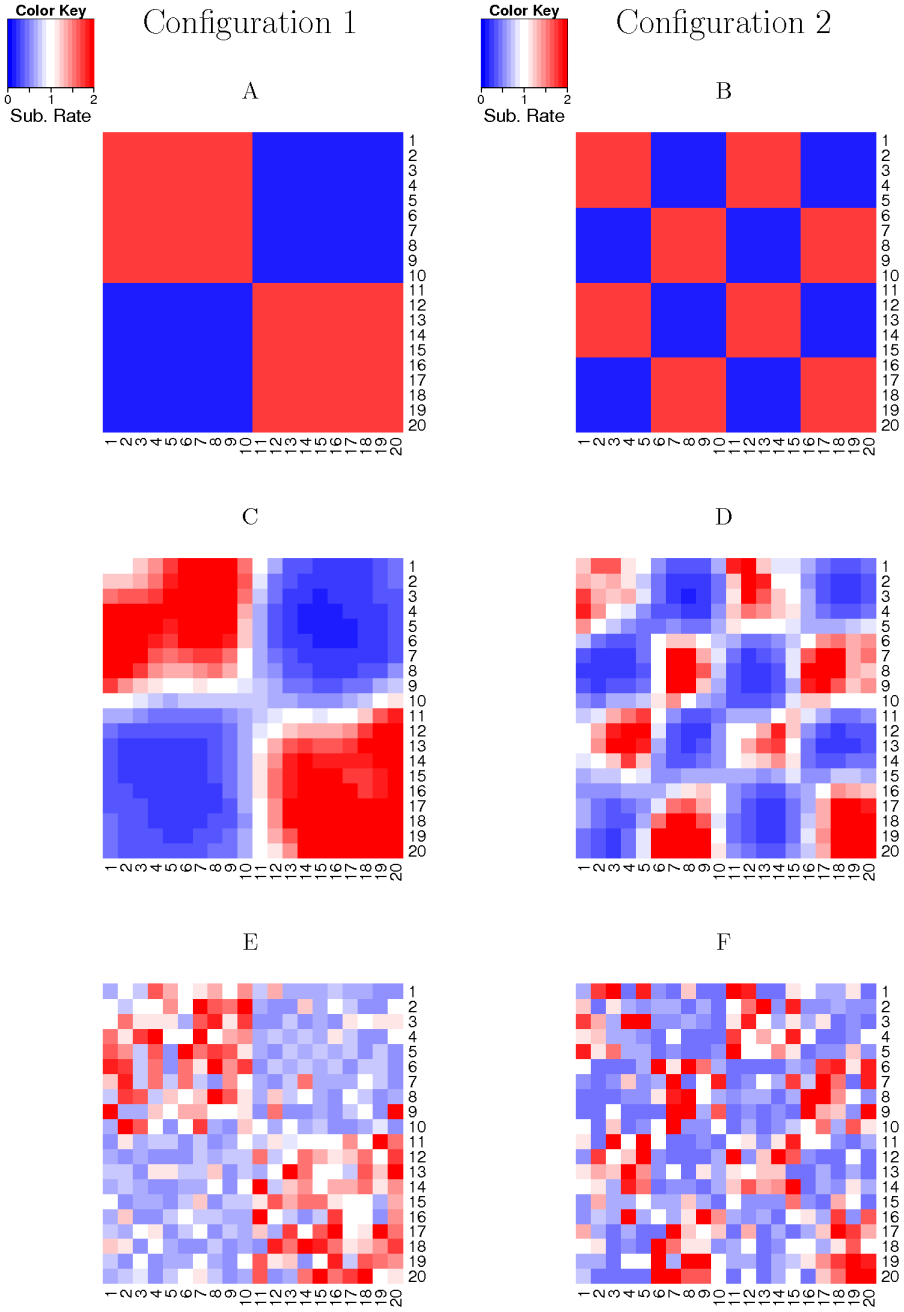
The visualization of the estimated site-specific substitution rates in the 2D toy protein simulations. The heatmaps are based on two randomly selected alignments, one for each configuration. The substitution rates in the heatmaps are arranged according to the toy 2D protein structure. (A, B) the true substitution rates in the first and second configurations, respectively; (C, D) the substitution rates estimated by GP4Rate in the first and second configurations, respectively; (E, F) the substitution rates estimated by Rate4Site in the first and second configurations, respectively.

In the 2D toy protein model, the protein tertiary structure was described by a 20 by 20 regular 2D grid, in which each dot corresponds to an amino acid in the toy protein structure (Figure 1B). In addition, we assumed that the distance between adjacent sites in the 2D grid is equal to 5 Å. This distance is comparable to the average distance between 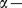
carbon atoms of the physically interacting residues in real proteins. Even though the 2D toy protein model is artificial and no real protein has a similar structure, it is useful because the estimated site-specific substitution rates can be easily visualized by a heatmap (Figure 2). Therefore, we used the 2D toy protein model to check the correctness of the program and to get insights on the performance of GP4Rate.

Two different spatial configurations of site-specific substitution rates were used in the 2D toy protein simulations. In the first configuration, the 20 by 20 grid was divided into 4 non-overlapping blocks, each of which was a 10 by 10 grid (Figure 2A). Sites within a block had the same substitution rates but different blocks could have different substitution rates. Two substitution rates, 0.2 and 1.8, were used for simulations and the substitution rates of blocks were alternatively arranged in the 2D protein structure (Figure 2A). Therefore, the toy proteins consisted of two conserved blocks with low substitution rates (0.2) and two variable blocks with high substitution rates (1.8). The second configuration was similar to the first one, but the sizes of non-overlapping blocks were 5 by 5 instead of 10 by 10 (Figure 2B). Twenty simulated alignments were generated for each configuration of site-specific substitution rates. It is easy to notice that the average site-specific substitution rate is equal to 1 in both configurations.

A program based on Bio++ [28], [29] was developed to implement the simulations. For each simulated alignment, we ran two separate MCMC chains using GP4Rate to estimate site-specific substitution rates. For each MCMC chain, 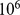 iterations were implemented and the trace plots of the MCMC outputs were monitored to ensure the convergence of the MCMC chains. The first 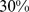 of the iterations were discarded as burn-in. Then, the two chains were combined to calculate the average substitution rate at each site. To compare the performance of GP4Rate with that of Rate4Site, we also used Rate4Site to estimate the substitution rates. To make the results of GP4Rate and Rate4Site more comparable, the phylogenetic tree and branch lengths were fixed to the true values in both GP4Rate and Rate4Site.

We firstly randomly sampled two simulated alignments, one for each configuration, as examples to get insights on the performances of GP4Rate and Rate4Site. As shown in Figure 2C and 2D, the site-specific substitution rates estimated by GP4Rate are smoothly distributed within the 2D protein structures. In addition, GP4Rate segments the 2D protein structures into blocks which correspond to the true patches with different substitution rates. In contrast, the spatial distributions of substitution rates estimated by Rate4Site are far from smooth. The sites with similar substitution rates are not clustered together and do not form clearly bounded patches (Figure 2E and 2F). Thus, GP4Rate can capture the spatial correlation of substitution rates but Rate4Site cannot.

### Quantitative evaluation of different models

**Figure 3:**
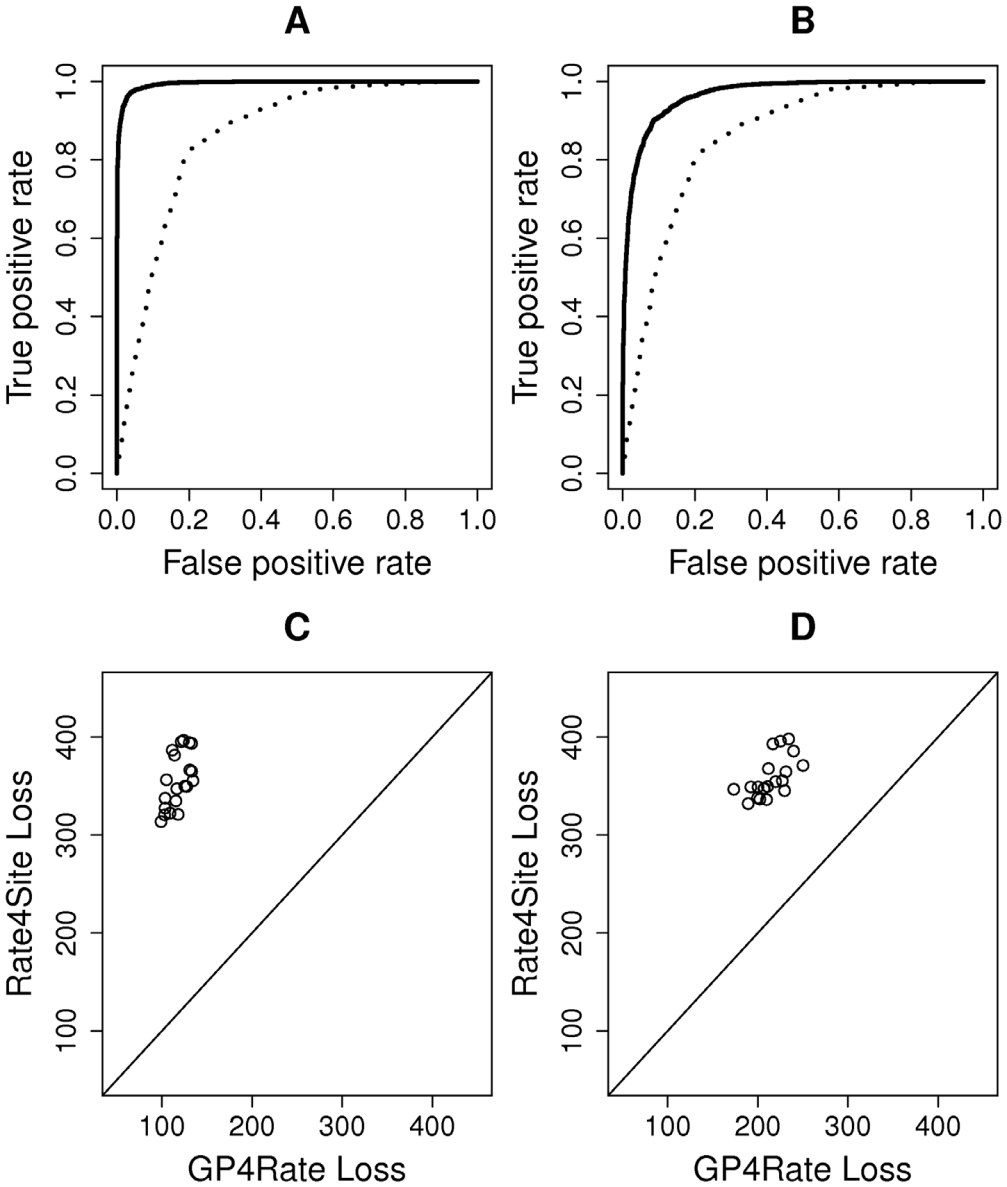
The quantitative comparison of GP4Rate and Rate4Site in the 2D toy protein simulations. (A) the ROC curves of GP4Rate and Rate4Site in the first configuration; (B) the ROC curves of GP4Rate and Rate4Site in the second configuration; (C) the losses of GP4Rate and Rate4Site in the first configuration; (D) the losses of GP4Rate and Rate4Site in the second configuration. In the ROC curves, the solid lines correspond to the performance of GP4Rate while the dotted lines correspond to the performance of Rate4Site. In the plots of losses, each point corresponds to a simulated alignment. The losses of the two methods are calculated by Equation 1.

To quantitatively evaluate the performance of GP4Rate and Rate4Site, we used receiver operating characteristic (ROC) curves to measure the power of the two methods. ROC curves are widely used to evaluate the accuracy of binary classifiers. The area under a ROC curve is usually used as a measure of the power of the corresponding method. To apply ROC curves to the simulated datasets, we must divide the amino acid sites into two categories, functional sites and nonfunctional sites, before generating simulated alignments. The functional sites are used as true positives while the nonfunctional sites are used as true negatives. In the 2D toy protein simulations, functional sites evolved at the lower rate (0.2) while nonfunctional sites evolved at the higher rate (1.8). Then, the ROC curves were created by plotting the average true positive rates *versus* the average false positive rates using the ROCR library in R [30]. As shown in Figure 3A and 3B, the areas under the ROC curves generated by GP4Rate are larger than those generated by Rate4Site, which suggests that GP4Rate outperforms Rate4site.

ROC curves measure whether a model can distinguish slowly evolved functional sites from the other sites. If a model can assign relatively low substitution rates to slowly evolved sites and relatively high rates to the other sites, it will perform well in the evaluations based on ROC curves. However, ROC curves cannot capture potential systematic biases of the model. For example, if the model adds a constant bias to the site-specific substitution rates, its ROC curves will be exactly the same regardless of the magnitude of the constant bias. Therefore, we used a simple loss function complementary with the ROC curves to capture any potential systematic biases of the estimated site-specific substitution rates. The loss function is defined by the following formula
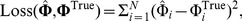
(1)in which 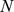 is the total number of sites in the alignment, while 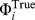 and 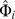 are the true and estimated log substitution rates at site *i*, respectively. The log values of site-specific substitution rates are used in the right-hand side of Equation 1, since we want to emphasize the differences between low substitution rates. It is desirable because both GP4Rate and Rate4Site were designed to detect conserved regions with low substitution rates. Unlike ROC curves, a model which introduces a larger systematic bias will have a higher average loss than a model which introduces a smaller bias.

We plotted the losses of both GP4Rate and Rate4Site in the 2D toy protein simulations. As shown in Figure 3C and 3D, GP4Rate outperforms Rate4Site, as evident by the lower losses produced by GP4Rate (paired Wilcoxon test, 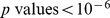 for both of the two configurations). The improved accuracy originates from GP4Rate's ability to model the spatial correlation of site-specific substitution rates, since the performance gap between GP4Rate and Rate4Site becomes smaller in the second configuration which consists of smaller conserved and variable patches.

**Figure 4:**
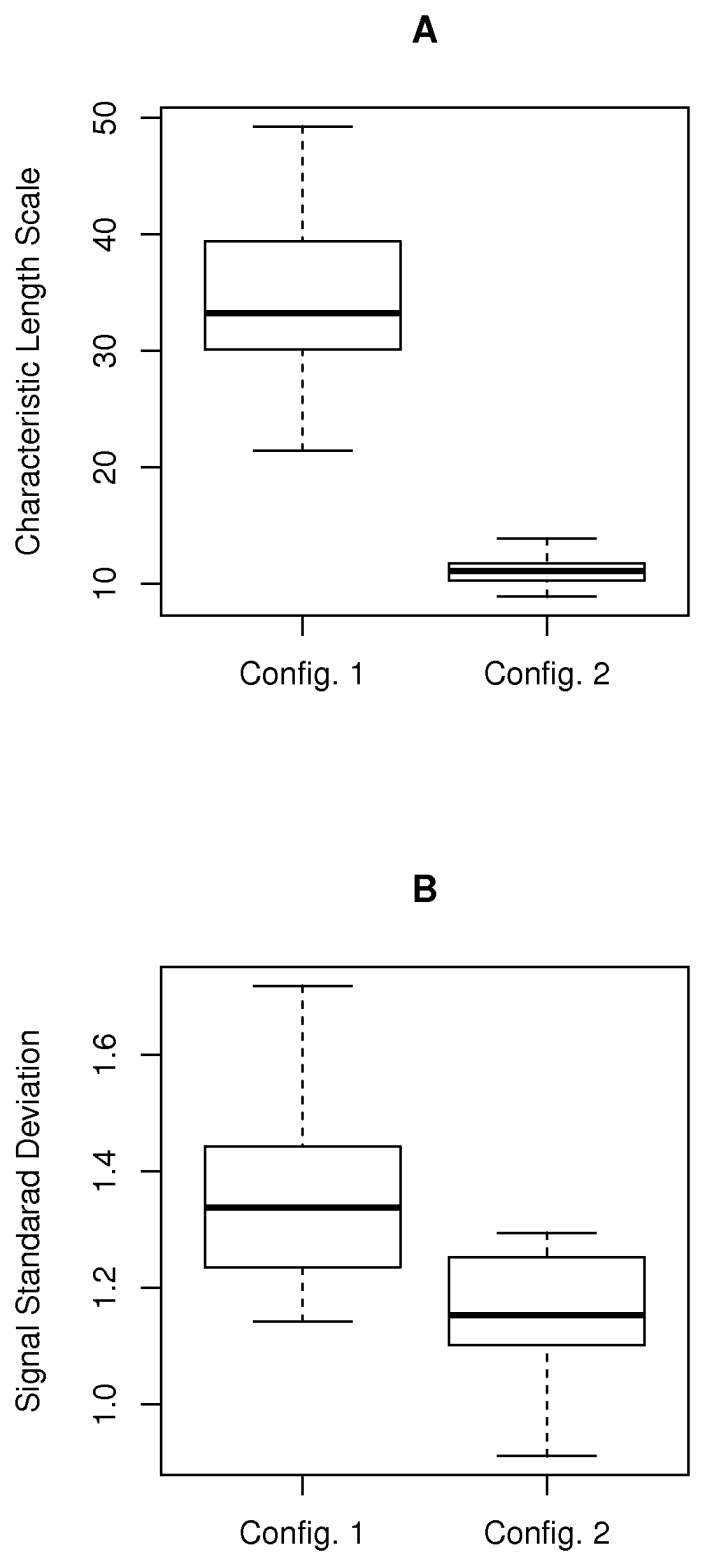
The hyperparameters estimated by GP4Rate in the 2D toy protein simulations. The unit of the characteristic length scale is Å while the signal standard deviation is unitless. (A) the estimated characteristic length scale; (B) the estimated signal standard deviation.

GP4Rate has two hyperparameters, i.e. the characteristic length scale 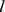 and the signal standard deviation 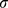, which model the strength of spatial correlation of substitution rates and the marginal variation of substitution rate at a single site, respectively. An advantage of GP4Rate over the sliding window methods is that the hyperparameters can be learned from the data. In contrast, the window size of the sliding window methods must be predefined before analyses. To show that GP4Rate can learn the hyperparameters from the data, we plotted the estimated median hyperparameters of the simulated alignments. As shown in Figure 4A, the characteristic length scales 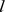 estimated in the first configuration are about 3 fold larger than those estimated in the second configuration. Because the patches are much larger in the first configuration, the result suggests that GP4Rate can learn the magnitude of the spatial correlation of substitution rates from the data. The estimated signal standard deviations 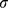 in the two configurations are similar, which matches the intuition that the two configurations are similar except in the strength of spatial correlations of substitution rates.

In summary, when spatial correlation of substitution rates exists in proteins, GP4Rate always outperforms Rate4Site. However, the spatial correlation of site-specific substitution rates may be insignificant in some proteins. Therefore, we also evaluated both GP4Rate and Rate4Site in simulated alignments in which the spatial correlation of site-specific substitution rates is absent. These simulated alignments were generated by randomly shuffling the columns in each alignment in the first spatial configuration of substitution rates (Figure 2A). The permutations of alignments destroyed the spatial patten of site-specific substitution rates. Here we only summarize the performance of GP4Rate and Rate4Site in the permuted alignments and more details can be found in the online Supplementary Material. The absence of spatial correlation results in close-to-zero characteristic length scales in GP4Rate, which confirms that GP4Rate can detect the absence of spatial correlation when there is none. Plots of ROC curves show that GP4Rate and Rate4Site have effectively the same power to distinguish slowly evolved sites from the other sites. In contrast, when we use the loss function (Equation 1) to measure the accuracy of estimated substitution rates, GP4Rate is less accurate than Rate4Site. Nevertheless, GP4Rate and Rate4Site have similar power to find slowly evolved functional sites, since in practice it is the relative rankings of sites instead of their absolute substitution rates tell us which sites may be more likely to be functional.

### Realistic simulations

We generated a second set of simulated alignments based on more realistic assumptions. The basic idea is that if we have a large number of highly diverged sequences, a simple method which does not consider the spatial correlation of substitution rates may accurately estimate the site-specific substitution rates because of the rich information in a very large dataset. We may generate simulated alignments based on the real protein tertiary structure and the presumably accurately estimated site-specific substitution rates. These simulated alignments may have similar features as real proteins.

In this set of simulations, we used the same phylogenetic tree (Figure 1A) and the JTT substitution model [26], [27] used in the 2D toy protein simulations. The protein tertiary structure and the site-specific substitution rates were based on a real protein, B-cell lymphoma extra large (Bcl-xL). This protein has been studied using Rate4Site and the two predicted conserved patches coincide with the regions with known functions [31]. We downloaded the protein tertiary structure of Bcl-xL from Protein Data Bank (PDB ID: 1MAZ [32]). The site-specific substitution rates estimated by Rate4Site were obtained from the ConSurf-DB database [10]. In ConSurf-DB, 131 unique homologs of Bcl-xL were automatically collected and then Rate4Site was applied to estimate the site-specific substitution rates. Because of the very large number of sequences in the dataset, the estimation of site-specific substitution rates may be relatively accurate. We generated 20 simulated alignments based on the above assumptions and both GP4Rate and Rate4Site were applied to the simulated alignments using the same setting described in the 2D toy protein simulations.

**Figure 5:**
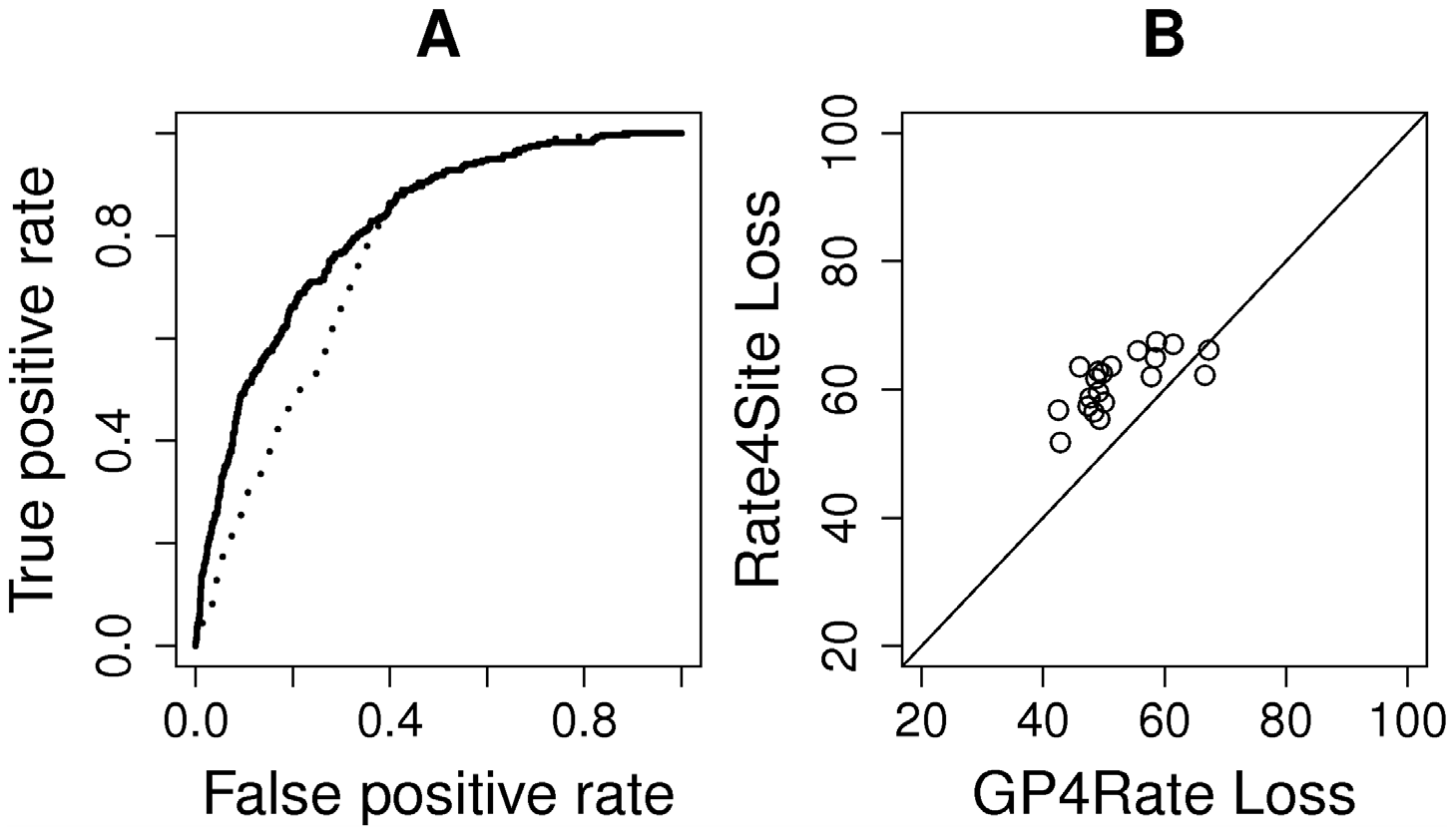
The quantitative comparison of GP4Rate and Rate4Site in the realistic simulations. (A) the ROC curves of GP4Rate and Rate4Site in the realistic simulations; (B) the losses of GP4Rate and Rate4Site in the realistic simulations. In the ROC curves, the solid line corresponds to the performance of GP4Rate while the dotted line corresponds to the performance of Rate4Site. In the plot of losses, each point corresponds to a simulated alignment. The losses of the two methods are calculated by Equation 1.

To evaluate the performance of GP4Rate and Rate4Site by ROC curves, we divided the sites into two categories before generating simulated alignments: slowly evolved functional sites and others. Based on the site-specific substitution rates reported by ConSurf-DB, the 10 percent most slowly evolved sites were considered to be functional while the others were not. As shown in Figure 5A, GP4Rate is more powerful to distinguish slowly evolved sites from the other sites, since the area under the ROC curve of GP4Rate is larger than that of Rate4Site. In addition, based on the loss function defined by Equation 1, GP4Rate produces lower losses in 18 out of the 20 simulated alignments (Figure 5B) and the median loss of GP4Rate is significantly smaller than that of Rate4Site (paired Wilcoxon test, 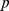 value<10^−4^). Therefore, GP4Rate still outperforms Rate4Site in the realistic simulations.

### Case study of B7-1 genes

The B7-1 (CD80) family is a member of the immunoglobulin superfamily (IgSF) and is critical for the regulation of immune responses [33]. The protein tertiary structure of the human B7-1 protein has been determined [34], [35]. The human B7-1 protein consists of two IgSF domains (IgV and IgC), each of which shows an anti-parallel 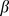 sandwich structure [34]. We applied GP4Rate and Rate4Site to 7 mammalian B7-1 sequences downloaded from the NCBI HomoloGene database [36] and compared their performances. The N-terminal and C-terminal sequences were trimmed in the alignment, because the corresponding atoms are absent in the X-ray crystal structure. The resulting alignment consists of 199 amino acid sites. Then the phylogenetic tree was inferred by PhyML with the 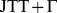 model [37]. The protein sequences in the alignment are very similar to each other as evident by the lack of gaps in the alignment (data not shown). Therefore, the information in each site in the alignment is very limited and it is hard to infer site-specific substitution rates accurately.

**Figure 6:**
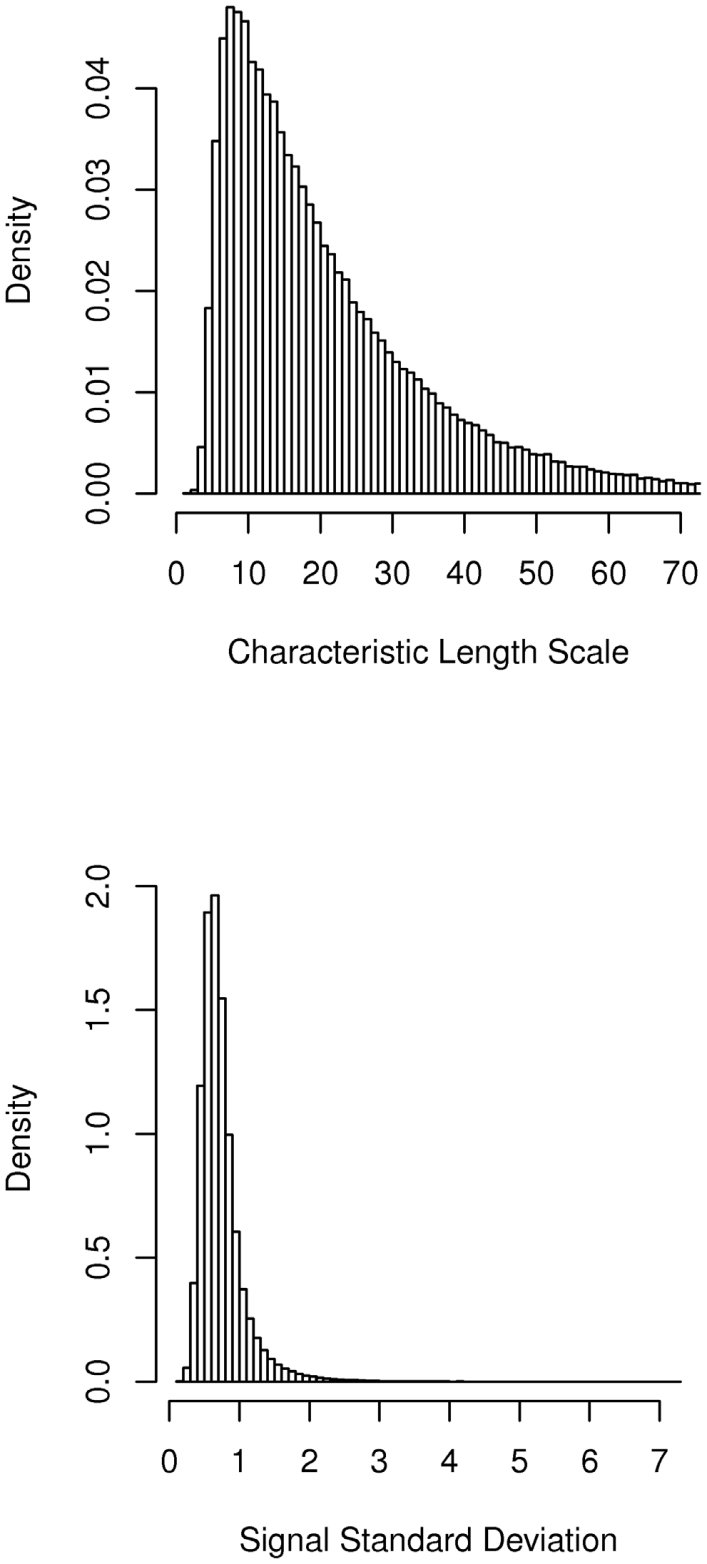
The empirical marginal density functions of the hyperparameters in the case study of B7-1 genes. The unit of the characteristic length scale is Å while the signal standard deviation is unitless.

**Figure 7:**
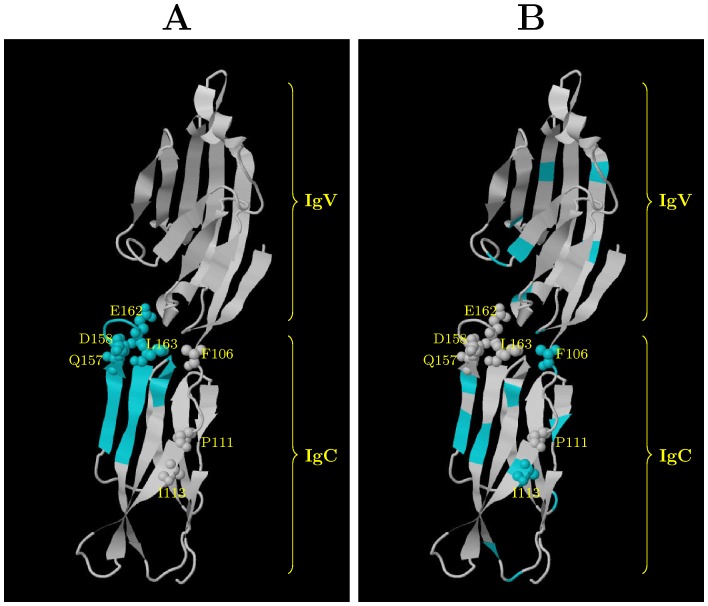
The locations of the 20 most conserved sites in the protein tertiary structure of the human B7-1 protein (PDB ID: 1I8L). The blue sites are the 20 most conserved sites and the space-filled atoms correspond to the experimentally verified functional sites in the IgC domain [38]. The experimentally verified functional sites in the IgV domain are not shown. The protein structures are visualized by Jmol [59]. A list of the most conserved sites can be found in the online Supplementary Material. (A) the 20 most conserved sites predicted by GP4Rate; (B) the 20 most conserved sites predicted by Rate4Site.

We used the human B7-1 protein structure (PDB ID: 1I8L [35]) to calculate the pairwise Euclidean distances between the 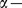
carbon atoms of amino acids. Then, we applied GP4Rate to the B7-1 alignment to infer site-specific substitution rates. We ran two independent MCMC chains for 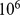 iterations, and the first 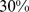 of the iterations were discarded as burn-in. We first estimated the posterior marginal distributions of hyperparameters based on the MCMC samples. As shown in Figure 6, the estimated characteristic length scale 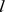 is significantly higher than 0, which confirms that the substitution rates are correlated in real proteins. The presence of spatial correlation of substitution rates may facilitate the discovery of slowly evolved functional regions. To test this hypothesis, the mean site-specific substitution rates of the MCMC samples were calculated and the 20 most slowly evolved sites were considered to be functional. Then, the 20 most slowly evolved sites were superimposed onto the protein tertiary structure (PDB ID: 1I8L [35]). As shown in Figure 7A, the slowly evolved sites predicted by GP4Rate are not randomly distributed and instead form a single large region in the IgC domain. A systematic mutagenesis study has suggested that the IgC domains are important for binding CTLA-4 and CD28 [38], even though the effects of the IgC domain may be indirect [35]. To test whether the predicted slowly evolved sites overlap with the experimentally verified functional sites [38], the 7 experimentally verified functional sites in the IgC domain were mapped onto the human B7-1 structure (Figure 7A). Clearly 4 experimentally verified functional sites in the IgC domain, i.e. Q157, D158, E162, and L163, are within the slowly evolved patch predicted by GP4Rate, which highlights the potential usefulness of GP4Rate.

To compare GP4Rate with Rate4Site, we also applied Rate4Site to the same dataset. The superimposition of the 20 most slowly evolved sites predicted by Rate4Site is shown in Figure 7B. The sites predicted by Rate4Site are present in both the IgV and IgC domains and do not form clearly bounded regions. Even though 2 experimentally verified functional sites in the IgC domain, i.e. F106 and I113, overlap with the sites predicted by Rate4Site, the 4 experimentally verified functional sites detected by GP4Rate do not overlap with the sites predicted by Rate4Site. Therefore, GP4Rate and Rate4Site can provide complementary insights to real data.

To investigate which model, GP4Rate or Rate4Site, fits the B7-1 dataset better, we performed a Bayesian model comparison. The direct comparison between GP4Rate and Rate4Site is impractical, because Rate4Site is based on the maximum likelihood principle instead of the Bayesian principle. However, it is not very difficult to develop a Bayesian version of Rate4Site by specifying a prior distribution over parameters. Therefore, we developed a Bayesian version of Rate4Site and compared it with GP4Rate. Details of the Bayesian model comparison can be found in the online Supplementary Material and we only summarize the results here. We compared the site-specific substitution rates estimated by the original Rate4Site and its Bayesian version and found that the two programs produced essentially the same result. Therefore, the marginal likelihood estimated by the Bayesian version of Rate4Site may be used to evaluate how good the original Rate4Site fits the B7-1 dataset. The log marginal likelihood of GP4Rate is equal to 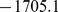 while the log marginal likelihood of the Bayesian Rate4Site is equal to 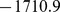, which suggests a very large Bayes factor of GP4Rate compared with the Bayesian Rate4Site (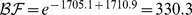). Therefore, GP4Rate fits the B7-1 dataset much better than the Bayesian Rate4Site.

## Discussion

Many phylogenetic methods have been developed to identify slowly evolved amino acid sites which may be functional. However, the most widely used methods, e.g. Rate4Site, ignore the spatial correlation of site-specific substitution rates. Some other methods use the sliding-window framework to capture the spatial correlation of substitution rates, but the statistical method for choosing the optimal window size is largely unknown. Since the strength of the spatial correlation of substitution rates is unknown in most of proteins, the sliding window methods are problematic in real data analyses. In GP4Rate, both of the two issues are solved under a Bayesian statistical framework. By using the Gaussian process to define the prior distribution of the site-specific log substitution rates, GP4Rate can naturally model the spatial clustering of functionally important sites and the hyperparameters which measure the strength of spatial correlation can be inferred from the data instead of being manually specified before the analyses.

In simulated datasets, GP4Rate significantly outperforms Rate4Site. The power of GP4Rate is mainly derived from the fact that GP4Rate has the added ability to model the spatial correlation of substitution rates. By borrowing statistical information from neighbor sites with similar substitution rates, GP4Rate can estimate the site-specific substitution rates with a much higher accuracy than Rate4Site. In the case study of B7-1 genes, GP4Rate predicted a slowly evolved functional patch in the protein tertiary structure and 4 sites within the region are well supported by experimental evidence. In contrast, the slowly evolved sites predicted by Rate4Site are scattered and do not form clearly bounded regions. In addition, we have shown that GP4Rate fits the B7-1 dataset much better than Rate4Site based on Bayesian model comparison.

The performance gap between GP4Rate and Rate4Site will be maximized when the protein sequences are very similar to each other and the spatial correlation is strong. Therefore, GP4Rate is most suitable to analyze small gene families, e.g. new genes or small gene families derived from recent gene duplication events. When the spatial correlation of substitution rates is weak, GP4Rate and Rate4Site may generate similar results. For example, we applied GP4Rate to 38 RH1 genes [39] and found that the spatial correlation of substitution rates is much weaker in the RH1 dataset than that in the B7-1 dataset (data not shown). In this case, the difference between GP4Rate and Rate4Site is subtle. Therefore, a rigorous model comparison as shown in the case study of B7-1 genes may be important in data analyses.

Because GP4Rate is based on MCMC simulations, it is slower than Rate4Site. For example, it took about 1 CPU day for GP4Rate to analyze the B7-1 dataset. However, GP4Rate is still very useful for small scale problems, e.g. guiding mutagenesis experiments, since the experimental time is much longer than the execution time of GP4Rate. The time cost of GP4Rate can be reduced in the future using advanced algorithms, e.g. more efficient MCMC sampling algorithms or sparse approximations of the Gaussian process [40]. The most time consuming step of GP4Rate is the Cholesky decomposition whose time complexity is a cubic function of the number of sites in the alignment. In practice, a simple method to reduce the computational time is to perform the analyses based on a selected subset of amino acid sites. For example, it is well known that surface residues are more likely to be involved in interactions with other proteins or ligands. If these interactions are most interesting to users, a fast analysis based only on the surface residues may be appropriate.

In addition to modeling the spatial correlation of site-specific substitution rates, protein tertiary structures have been used to improve phylogenetic models and the estimation of site-specific substitution rates in a few other studies [41]–[46]. These methods can be roughly divided into two categories. The first category of models assumes that the fixation probability of new mutations is determined by how the mutations influence the stability of the protein [41]–[43]. Typically it is assumed that mutations which stabilize the protein structure are more likely to be fixed than mutations which destabilize the protein structure. Unlike this category of models, the Phylo-GPM framework does not provide a mechanistic interpretation for the estimated substitution rates. However, GP4Rate may be more powerful to identify functional regions which are not directly relevant to the stability of proteins. The second category of models assumes that the site-specific substitution rates or *dN/dS* ratios are influenced by the local environment of the focal site in the protein tertiary structure [44]–[46]. For example, it has been shown that the *dN/dS* ratio of a site is influenced by its relative solvent accessibility (RSA) [44]–[46]. It is relatively straightforward to combine the Phylo-GPM framework with local features of amino acid sites. For example, in this study we assume that the site-specific log substitution rates follow a zero-mean Gaussian distribution. We may replace the zero-mean rate vector by a new one in which the mean of log substitution rate at a site is a linear function of its local features, e.g. RSA. It is very interesting to investigate whether adding local features to the Phylo-GPM framework improves model fitting in the future.

The Phylo-GPM framework proposed in this paper may be used as a general tool to model the spatial correlation of patterns in the protein tertiary structure. The phylogenetic hidden Markov model (Phylo-HMM) is a popular method which combines the hidden Markov model and statistical phylogenetics [47]. It has been used to model the spatial correlation of evolutionary patterns along primary sequences [17], [48]–[53]. The Phylo-GPM framework may be viewed as an extension of the Phylo-HMM to the protein tertiary structures. In the future, new methods based on the Phylo-GPM framework may be developed to identify functional divergence or positive selection in proteins.

## Models

### Overall design of the phylogenetic Gaussian process model

GP4Rate is an open-source software application written in C++ and its source code is freely available from http://info.mcmaster.ca/yifei/software.html. GP4Rate combines the protein alignment and the protein tertiary structure to infer groups of close-located functional sites evolved at low rate. We assume that the protein alignment, the phylogenetic tree, and the tertiary structure of one protein in the alignment are provided by users. In GP4Rate, both the topology and the branch lengths of the phylogenetic tree are fixed to improve the speed of the program. In addition, we assume that the protein sequences in the alignment belong to the same gene family and have very similar functions, which implies that the functionally important sites do not vary among sequences and the site-specific substitution rates do not change over time. However, we do assume that the substitution rates can vary across different sites. The site-specific rates are used as proxies of functionality: very low substitution rates suggest the corresponding sites are functionally important.

In most molecular phylogenetic programs, e.g. Rate4Site [7], PAML [54], and PhyML [37], the site-specific substitution rates are assumed to be i.i.d. and follow a simple discrete distribution, usually the discrete Gamma distribution [25]. Recently, Dirichlet process pirors have been used to model the variable substitution rates over sites to overcome the inflexibility of the simple discrete distributions [55], but it is still assumed that the site-specific substitution rates are i.i.d. The i.i.d. assumption implies that slowly evolved functional sites are scattered in the protein tertiary structure. The major contribution of this paper is to relax the i.i.d. assumption using the Gaussian process [21] which can naturally capture the spatial correlation of site-specific substitution rates in the protein tertiary structure.

In GP4Rate, the parameters are estimated using the Bayesian principle. In Bayesian statistics, the parameters are random variables and the conditional distribution of parameters given data, i.e. the posterior distribution, gives us an estimation of parameters. For simplicity of presentation, first we focus on the vector of site-specific log substitution rates, which is the collection of log values of substitution rates at all amino acid sites, and defer the discussions on the other parameters. The posterior distribution of the vector of log site-specific substitution rates can be defined by the following equation,
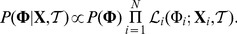
(2)In the equation, 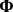 is the vector of site-specific log substitution rates, 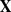 is the protein alignment while 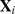 is its *i*-th column, and 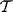 is the phylogenetic tree with the associated branch lengths. 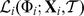 is the site-specific likelihood at site *i*, which is a function of the site-specific log substitution rate at site *i*. 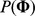 is the fundamentally important prior distribution of site-specific log substitution rates.

A realistic 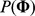 should be able to describe the spatial correlation of site-specific substitution rates. In GP4Rate, 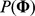 is specified by a zero-mean Gaussian process. A Gaussian process is a probability measure defined over a function space. In the statistical modeling of site-specific substitution rates, we are only interested in the marginal distribution of the Gaussian process over a finite set of spatial locations which correspond to the locations of residues in the protein tertiary structure. By the definition of Gaussian processes, the marginal distribution of a zero-mean Gaussian process is a zero-mean multivariate Gaussian distribution [21]. Therefore, 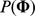 may be rewritten in the following format,
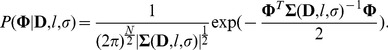
(3)The correlation of site-specific substitution rates is determined by the covariance matrix 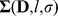, in which 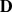 is the pairwise distance matrix which measures the Euclidean distance between the 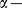
carbon atoms of amino acids in the protein tertiary structure. Furthermore, the covariance function is parameterized by two hyperparameters, 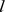 and 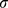, which measure the strength of spatial correlation and the variation of substitution rates across sites, respectively. By plugging 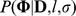 and 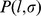, the prior distribution of the hyperparameters, into Equation 2, it can be expanded to the following format,
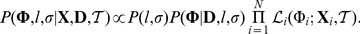
(4)In the following sections, we will provide more details on the specifications of the right-hand side terms of Equation 4 and the MCMC algorithm for the sampling of parameters, i.e. 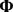, 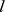, and 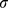.

### Gaussian process as a prior distribution of site-specific log substitution rates

As mentioned above, 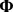 follows a zero-mean multivariate Gaussian distribution (Equation 3). In the multivariate Gaussian distribution, the covariance matrix 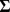 is specified by a covariance function. By default, GP4Rate uses the Matérn 1.5 covariance function,
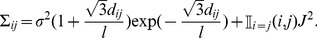
(5)In the equation, 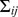 is an element in the covariance matrix 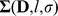 while 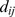 is an element in the distance matrix 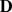 which measures the Euclidean distance between site *i* and site *j* in the protein tertiary structure. 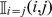 is an indicator function which is equal to 1 if site *i* and site *j* are the same site and equal to 0 otherwise. The covariance function contains two free parameters, 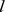 and 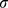. 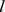 is the characteristic length which determines the strength of the spatial correlation of substitution rates. If it is small, the spatial correlation is weak and only nearby sites have similar log substitution rates. Instead, if it is large, the spatial correlation is strong and distant sites can have similar log substitution rates. 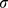 is the signal standard deviation which measures the marginal variation of log substitution rates at a single site. Small 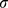 implies that the variation of log substitution rates is small. 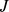 is a fixed “jitter” term which introduces a small amount of noise to the diagonal elements in 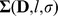. The “jitter” term ensures that the Cholesky decomposition, a critical numerical algorithm in the MCMC simulations, is numerically stable and improves the mixing of the MCMC simulations [56]. The “jitter” term is usually a small positive number (e.g. 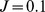), so it does not significantly change the behavior of the covariance function [56]. Clearly Equation 5 implies that the covariance of log substitution rates are decreasing with increasing Euclidean distance between two amino acid sites, which is compatible with our intuition that nearby sites tend to have similar substitution rates due to similar functions.

In addition to the Matérn 1.5 covariance function, GP4Rate has two alternative covariance functions for users to choose. One is the Matérn 2.5 covariance function,
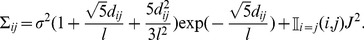
(6)The other is the widely used squared-exponential covariance function,
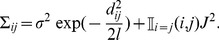
(7)

The three covariance functions are all special cases of the general Matérn covariance function [21]. The major difference between them is that the three covariance functions describe different levels of smoothness in the spatial distribution of site-specific log substitution rates [21]. In the squared-exponential covariance function, the site-specific log substitution rates are smoothly distributed in the protein tertiary structure. Therefore, it is most suitable to model proteins with relatively large functional regions. In contrast, the Matérn 1.5 covariance function is the least smooth one and is suitable to model proteins with small functional patches. In this paper, we used the Matérn 1.5 covariance function in all analyses to allow for proteins that may have relatively small functional patches and could have nearby sites with very different substitution rates.

The hyperparameters in the covariance functions, i.e. 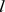 and 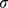, follow a prior distribution 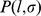. We assume that the characteristic length, 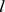, and the signal standard deviation, 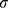, are independent and follow exponential distributions. Therefore, the prior distribution is defined by the following probability density function,
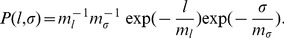
(8)We choose 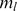 and 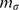 to be large so that the prior distribution has relatively weak information.

### Approximation of the phylogenetic likelihood function

To fully define the unnormalized posterior distribution (Equation 4), the likelihood 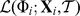 must be specified. We follow the standard phylogenetic model first described by Felsenstein [22]. We assume that the substitution model in the phylogenetic likelihood function is fixed to the JTT model [26], [27] while the phylogenetic tree is fixed to the one provided by the users. The likelihood can be calculated by the pruning algorithm and the gaps in the alignment may be treated as missing data [22]. However, the calculation of the likelihood function can easily become the most time consuming step in the MCMC sampling, because we need to evaluate the likelihood millions of times. We have applied a simple linear interpolation method to reduce the computational time of the likelihood evaluation [57]. GP4Rate calculates the site-specific log likelihoods at a set of evenly spaced substitution rates and then approximates the site-specific log likelihoods at other rates by interpolation. Note that the linear interpolation is performed based on the site-specific substitution rates while 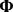 is the vector of their log values, so an exponential transformation, i.e. 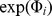, must be performed for each site *i* before the interpolation. By default, GP4Rate calculates and caches the site-specific log likelihoods at 4000 evenly spaced substitution rates, ranging from 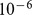 to 20. In each step of the likelihood calculation, if 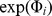 is between 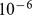 and 20, the corresponding site-specific log likelihood is approximated by the following formula,
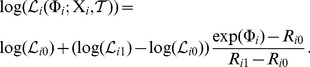
(9)On the right hand side, 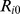 and 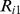 are the two cached substitution rates which are closest to 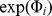, while 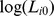 and 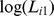 are the site-specific log likelihoods of 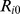 and 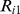, respectively. In practice, 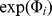 is rarely bigger than 20 or smaller than 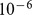. If it is indeed outside this, the log likelihood at the closest boundary is used as the approximate log likelihood.

### Markov Chain Monte Carlo sampling

GP4Rate uses MCMC simulations to sample parameters from their posterior distribution. The algorithm follows previous studies by Neal [56], [58]. As described in the previous sections, the parameters in GP4Rate have two components. The first one is 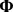 and the second one consists of 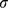 and 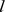. In each iteration, the two components are sequentially updated by the Metropolis algorithm with symmetric proposals [23], [24].

To update 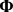, GP4Rate uses a proposal distribution suggested by Neal [56],
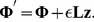
(10)In the equation, 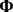 is the current vector of site-specific log substitution rates while 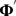 is the new proposal. 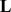 is the Cholesky decomposition of the covariance matrix 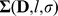 and 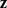 is a vector of independent standard Gaussian variables. The proposal distribution is tuned by the constant, 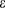. A large 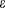 leads to large changes of 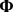 while small 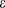 leads to small changes. 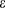 is chosen to make the acceptance rate of new proposals close to 0.25.

Instead of updating 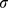 and 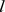 in the original scale, we transform them to the log scale. The use of a log scale removes the boundaries of the two parameters and makes the MCMC sampling of 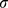 and 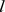 independent from the scale of the data [56]. The two parameters are updated by a sliding window method with a bivariate Gaussian proposal [58]. The Gaussian proposal is tuned so that the acceptance rate of new proposals is close to 0.25.

In practice, the update of 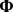 is much faster than the update of 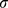 and 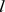, because the update of 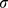 and 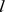 requires a Cholesky decomposition whose time complexity is 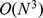, in which 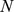 is the total number of sites in the alignment. Therefore, it is reasonable to update 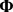 more often than 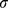 and 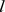 [56]. In each iteration 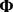 is updated 50 times while the pair of 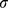 and 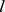 is updated once. After every 10 iterations, the values of 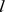, 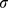, and 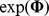 are recorded.

## Supporting Information

Table S1 **The list of most slowly evolved sites predicted by GP4Rate and Rate4Site in the case study of B7-1 genes.**
(XLS)

Text S1 **The comparison of GP4Rate and Rate4Site in the simulations without spatial correlation of substitution rates and the Bayesian model comparison of GP4Rate and Rate4Site in the case study of B7-1 genes.**
(PDF)
