# Supplementary material for: Phylogenetic Gaussian Process Model for the Inference of Functionally Important Regions in Protein Tertiary Structures
Source: PLoS Comput Biol. 2014 Jan 16;10(1):e1003429. doi: 10.1371/journal.pcbi.1003429 (PMC3894161; doi:10.1371/journal.pcbi.1003429)
Supplement: Text S1 — The comparison of GP4Rate and Rate4Site in the simulations without spatial correlation of substitution rates and the Bayesian model comparison of GP4Rate and Rate4Site in the case study of B7-1 genes. (PDF) [file pcbi.1003429.s002.pdf]

Supplementary Results

for

Phylogenetic Gaussian Process Model for the Inference of Functionally Important Regions in Protein Tertiary Structures

Yi-Fei Huang and G. Brian Golding

Department of Biology, McMaster University

2D toy protein simulations in the absence of the spatial correlation of site-specific substitution rates

Because the strength of spatial correlation of site-specific substitution rates may be very weak in some protein families, we compared the performance of GP4Rate and Rate4Site in simulated alignments in which the spatial correlation of site-specific substitution rates is absent. The simulated alignments were generated by randomly permuting the sites in each alignment in the first spatial configuration of the 2D toy protein simulations. The random permutations destroyed the spatial correlation of site-specific substitution rates but kept the other features of the data. We applied both GP4Rate and Rate4Site to the permuted alignments following the settings described in the Main Text. Because the spatial correlation of site-specific substitution rates is absent in these permuted alignments, we expected that the characteristic length scales estimated by GP4Rate would be very close to zero. As shown in Figure S1A, the estimated characteristic length scales are indeed close to zero. The result suggests that GP4Rate can detect the absence of spatial correlation of substitution rates in the permuted alignments.

Because GP4Rate is mainly designed for identifying slowly evolved functional sites in the presence of spatial correlation of substitution rates, it is interesting to test whether it has a similar statistical power as Rate4Site, which explicitly assumes the absence of spatial correlation, in the permuted alignments. Therefore, we plotted the ROC curves to visualize the performance of GP4Rate and Rate4Site. Similar to the 2D toy protein simulations described in the Main Text, we divided the sites into two categories, functional sites and nonfunctional sites, and these two categories were used as true positives and true negatives, respectively, in the ROC curves. The sites that evolved at the lower rate (0.2) were considered to be functional where these that evolved at the higher rate (1.8) were considered to be nonfunctional. As shown in Figure S2A, GP4Rate and Rate4Site have similar powers as the areas under the ROC curves of GP4Rate and Rate4Site are effectively identical.

As mentioned in the Main Text, ROC curves may not be able to estimate the potential systematic bias of the estimated substitution rates. Therefore, we com-

pared GP4Rate with Rate4Site using the simple loss function proposed in the Main Text. As shown in Figure S2B, GP4Rate has a lower accuracy than Rate4Site. The higher systematic bias in GP4Rate might be due to the inflexibility of the Gaussian process prior when a spatial correlation is absent. If the spatial correlation of substitution rates is absent, the estimated characteristic length scale will be very close to zero. In this scenario, the site-specific substitution rates are effectively independent and identically distributed (i.i.d) and the Gaussian process prior degenerates to a simple isotropic multivariate Gaussian distribution. Recalling that we assumed that the log values of site-specific substitution rates follow the Gaussian process prior, it means that the site-specific substitution rates effectively follow i.i.d. log-normal distributions. In contrast, Rate4Site assumes that site-specific substitution rates follow i.i.d. discrete Gamma distributions. It is well-known that Gamma distribution is very flexible and can model a variety of distributions with different shapes. In contrast, the log-normal distribution is not as flexible as the Gamma distribution. Nevertheless, in the practice of identifying functional sites, the absolute substitution rates are rarely interesting to researchers, since it is the relative substitution rates that tell us which sites may be functionally important. Because the ROC curves are equivalent between GP4Rate and Rate4Site, GP4Rate should have the same power as Rate4Site for identifying conserved functional sites if the spatial correlation of substitution rates is absent.

Bayesian model comparision in the case study of B7-1 genes

As mentioned in the main text, it is impractical to compare GP4Rate with Rate4Site directly, since GP4Rate is based on the Bayesian principle while Rate4Site is based on the maximum likelihood principle. Therefore, we developed a Bayesian version of Rate4Site. Because we assumed that both the topology and branch lengths of the phylogenetic tree were fixed in analyses, the only free parameter in Rate4Site is the shape parameter of the discrete Gamma distribution. In the Bayesian Rate4Site, we

assumed that the Gamma shape parameter follows a uniform distribution ranging from 0.05 to 5. The lower boundary was set to 0.05, because very small Gamma shape parameters, which suggest very large variations of site-specific substitution rates, are very unlikely to fit real data well and the discrete Gamma distribution is numerically instable when the Gamma shape parameter is very close to 0. The upper boundary, 5, corresponds to the scenario in which the variation of substitution rates is very small. Because there is only one free parameter in the Bayesian Rate4Site, we numerically integrated it out to calculate the log marginal likelihood of the Bayesian Rate4Site. More specifically, in the numerical integration we divided the range of the Gamma shape parameter into small bins whose sizes are all equal to 0.01. The marginal likelihood may be calculated by the following formula,

$$\mathcal{ML} = \frac{\sum_{i=1}^{K} \mathcal{L}_i^{\mathrm{Mid}}}{K}. \tag{S1}$$

In the equation, K is the total number of bins in the numerical integration while $\mathcal{L}_i^{\mathrm{Mid}}$ is the phylogenetic likelihood when the Gamma shape parameter is equal to the middle-point of the i-th bin. The site-specific substitution rates were also calculated using the same numerical integration algorithm.

To test whether Rate4Site and its Bayesian version lead to similar estimations of the site-specific substitution rates, we applied both the two programs to the B7-1 dataset described in the main text. As shown in Figure S3, the correlation of estimated site-specific substitution rates is very strong ($\rho > 0.999$). Therefore, the two programs generated essentially the same result and we may use the estimated log marginal likelihood of the Bayesian Rate4Site to measure how good the original Rate4Site fits the B7-1 dataset.

To calculate the log marginal likelihood of GP4Rate, we applied the steppingstone sampling (SS) algorithm [1]. It has been shown that the SS algorithm is a very accurate algorithm to calculate the log marginal likelihood of phylogenetic models [1]. The SS algorithm calculates the log marginal likelihood by performing a series of

MCMC simulations based on a family of distributions,

$$P(\mathbf{\Phi}, l, \sigma | \mathbf{X}, \mathbf{D}, \mathcal{T}, \beta) \propto P(l, \sigma) P(\mathbf{\Phi} | \mathbf{D}, l, \sigma) \{ \prod_{i=1}^{N} \mathcal{L}_i(\Phi_i; \mathbf{X}_i, \mathcal{T}) \}^{\beta}. \tag{S2}$$

The extra parameter β reflects the "temperature" of the system. If $\beta = 0$, we essentially sample from the prior distribution. If $\beta = 1$, we essentially sample from the posterior distribution. We choose 21 β values which correspond to the quantiles of the Beta$(0.3, 1)$ distribution as suggested by the previous study [1]. Then, 20 simulations were performed based on the chosen β values, each of which ran 10^6 iterations. The first 30% of samples were discarded as burn-in. Finally, the log marginal likelihood was calculated based on the 20 simulations [1].

The estimated log marginal likelihood of GP4Rate is equal to -1705.1 while the estimated log marginal likelihood of the Bayesian Rate4Site is equal to -1710.9. Recall that the Bayes factor is defined as the ratio of the marginal likelihoods of the two alternative models. The Bayes factor of GP4Rate compared with the Bayesian Rate4Site is equal to

$$\mathcal{BF} = e^{-1705.1+1710.9} = 330.3, \tag{S3}$$

which is significantly greater than 1. Therefore, GP4Rate fits the B7-1 dataset much better than the Bayesian version of Rate4Site.

References

[1] Xie W, Lewis PO, Fan Y, Kuo L, Chen MH (2011) Improving marginal likelihood estimation for Bayesian phylogenetic model selection. Systematic Biology 60: 150–160.

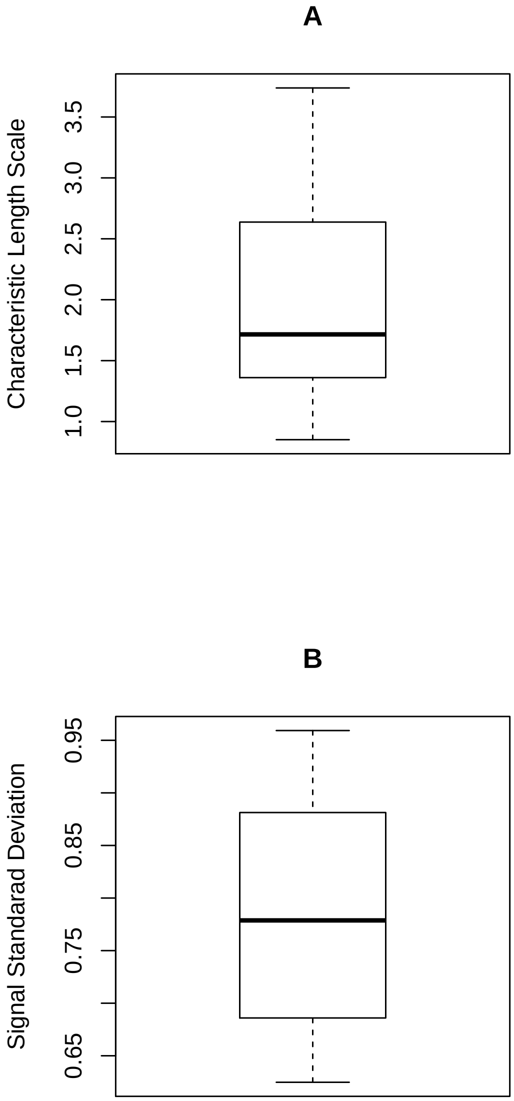

Figure S1: **The hyperparameters estimated by GP4Rate in the 20 permuted alignments.** The unit of the characteristic length scale is Å while the signal standard deviation is unitless. (A) the estimated characteristic length scale; (B) the estimated signal standard deviation.

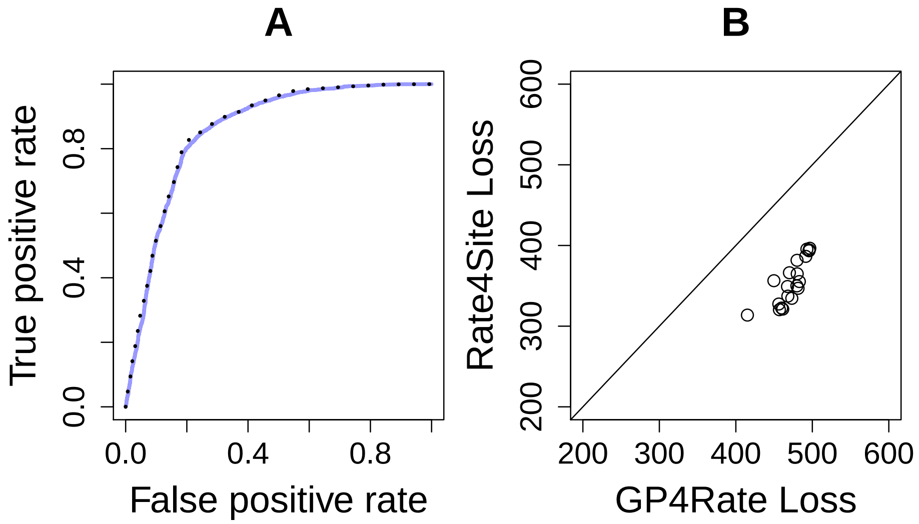

Figure S2: **The quantitative comparison of GP4Rate and Rate4Site in the 20 permuted alignments.** (A) the ROC curves of GP4Rate and Rate4Site; (B) the losses of GP4Rate and Rate4Site. In the ROC curves, the solid blue line corresponds to the performance of GP4Rate while the dotted black line corresponds to the performance of Rate4Site. In the plot of losses, each point corresponds to a permuted alignment.

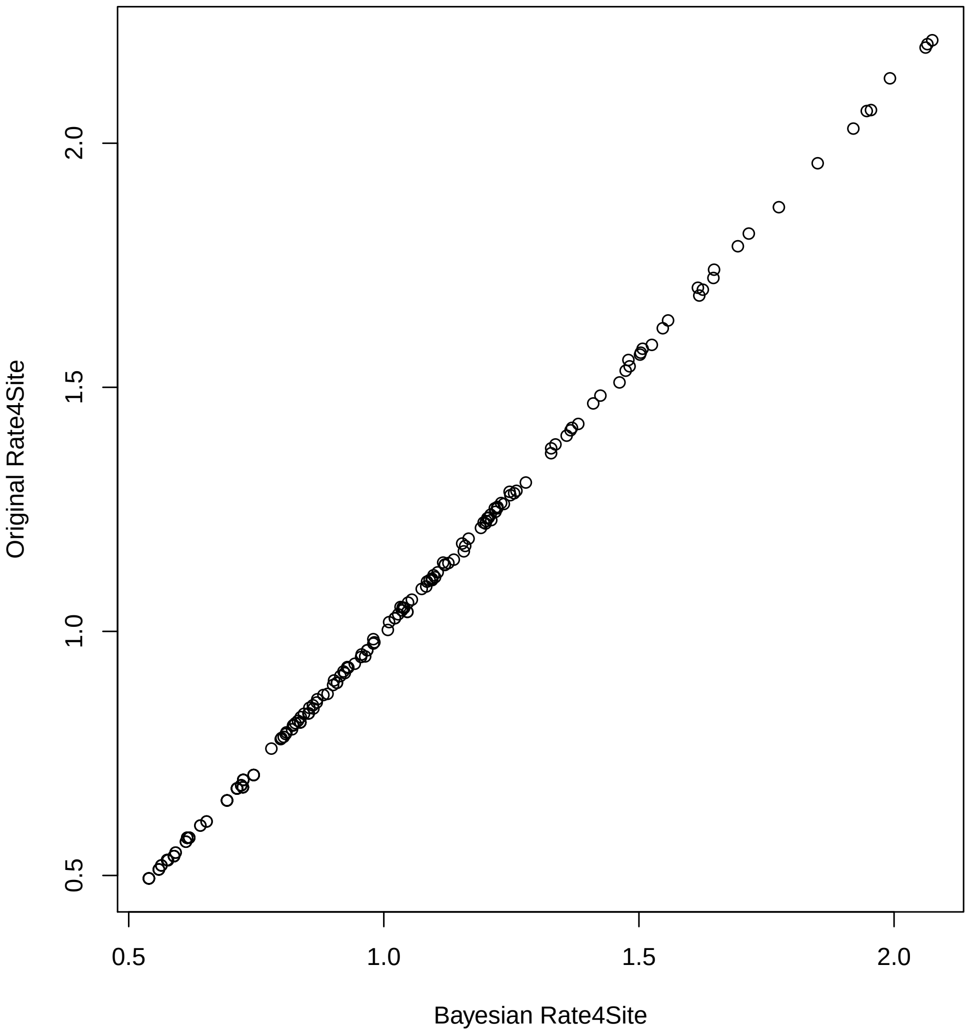

Figure S3: **The site-specific substitution rates estimated by Rate4Site and its Bayesian version in the case study of B7-1 genes.** The x-axis corresponds to the site-specific substitution rates estimated by the Bayesian Rate4Site while the y-axis corresponds to the site-specific substitution rates estimated by the original Rate4Site. The Spearman correlation coefficient of the estimated substitution rates is greater than 0.999.
